# Cotransplantation of Adipose Tissue-Derived Insulin-Secreting Mesenchymal Stem Cells and Hematopoietic Stem Cells: A Novel Therapy for Insulin-Dependent Diabetes Mellitus

**DOI:** 10.4061/2010/582382

**Published:** 2010-12-20

**Authors:** A. V. Vanikar, S. D. Dave, U. G. Thakkar, H. L. Trivedi

**Affiliations:** ^1^Department of Pathology, Laboratory of Medicine, Transfusion Services and Immunohematology, Dr. H. L. Trivedi Institute of Transplantation Sciences, India; ^2^Department of Nephrology and Transplantation Medicine, G. R. Doshi and K. M. Mehta Institute of Kidney Diseases and Research Centre (IKDRC), India; ^3^Dr. H. L. Trivedi Institute of Transplantation Sciences (ITS), Civil Hospital Campus, Asarwa, Gujarat, Ahmedabad 380016, India

## Abstract

*Aims*. Insulin dependent diabetes mellitus (IDDM) is believed to be an autoimmune disorder with disturbed glucose/insulin metabolism, requiring life-long insulin replacement therapy (IRT), 30% of patients develop end-organ failure. We present our experience of cotransplantation of adipose tissue derived insulin-secreting mesenchymal stem cells (IS-AD-MSC) and cultured bone marrow (CBM) as IRT for these patients. *Methods*. This was a prospective open-labeled clinical trial to test efficacy and safety of IS-AD-MSC+CBM co-transplantation to treat IDDM, approved by the institutional review board after informed consent in 11 (males : females: 7 : 4) patients with 1–24-year disease duration, in age group: 13–43 years, on mean values of exogenous insulin requirement of 1.14 units/kg BW/day, glycosylated hemoglobin (Hb1Ac): 8.47%, and c-peptide levels: 0.1 ng/mL. Intraportal infusion of xenogeneic-free IS-AD-MSC from living donors, subjected to defined culture conditions and phenotypically differentiated to insulin-secreting cells, with mean quantum: 1.5 mL, expressing Pax-6, Isl-1, and pdx-1, cell counts: 2.1 × 10^3^/*μ*L, CD45^−^/90^+^/73^+^:40/30.1%, C-Peptide level:1.8 ng/mL, and insulin level: 339.3  IU/mL with CBM mean quantum: 96.3 mL and cell counts: 28.1 × 10^3^/*μ*L, CD45^−^/34^+^:0.62%, was carried out. *Results*. All were successfully transplanted without any untoward effect. Over mean followup of 23 months, they had a decreased mean exogenous insulin requirement to 0.63 units/kgBW/day, Hb1Ac to 7.39%, raised serum c-peptide levels to 0.38 ng/mL, and became free of diabetic ketoacidosis events with mean 2.5 Kg weight gain on normal vegetarian diet and physical activities. *Conclusion*. This is the first report of treating IDDM with insulin-secreting-AD-MSC+CBM safely and effectively with relatively simple techniques.

## 1. Introduction

The incidence of diabetes mellitus (DM) has been increasing in an epidemic-like fashion in the last two decades globally. India is expected to become the world capital of DM by year 2030 [1–3]. Insulin dependent diabetes mellitus (IDDM) is the second most common chronic disease of childhood believed to be autoimmune in nature and characterized by irreversible destruction of insulin-secreting pancreatic *β* islet cells. Symptoms of the disease appear when insulin-making *β* cell mass gets reduced by approximately 90% leading to severe insulin deficiency and hyperglycemia. At present the only therapeutic options for management are life-long exogenous insulin preparations. Sporadic reports of autologous hematopoietic stem cell transplantation (HSCT) have been reported with limited success [4]. 

We present our experience of insulin replacement therapy (IRT) by co-transplantation of insulin-secreting adipose tissue derived mesenchymal stem cells (IS-AD-MSC) and cultured-bone-marrow- (CBM-) derived HSCT in 11 IDDM patients.

## 2. Study Design (Figure 1)

This was a prospective nonrandomized open-label clinical trial conducted from October 2007 to September 2008 to test the efficacy and safety of combined IS-AD-MSC and HSCT as IRT in IDDM patients. HSC co-transplantation with IS-AD-MSC was designed to augment the effect of the later. Omental vein infusion was carried out so that the cells would get trapped in hepatic microcirculation and the liver, being tolerogenic organ, would not reject them. The institutional Review Board approved of consent forms and clinical trial. 

Inclusion criteria were patients between 5 to 45 years of age, of any gender, with confirmed diagnosis of IDDM at least for 6 months, with low levels of serum C-peptide levels (<0.5 ng/mL).

Exclusion criteria were positive serology for HIV/HbSAg/HCV and underlying hematologic, nephrologic, cardiac, psychiatric, or hepatic diseases, and pregnancy.

Healthy nondiabetic donors from family of recipients having matching blood group with patients, who were willing to donate fat and bone marrow (BM) were approved as donors in this research protocol after their informed written consent.

## 3. Methods

### 3.1. Adipose Tissue and BM Procurement from Donor

Adipose tissue (approximately 2 gm) was resected from anterior abdominal wall of donors on day −14, sutures taken after hemostasis were achieved and sent to stem cell lab for culture in appropriate transport medium to derive MSC and further differentiate them into insulin-secreting cells. On days −10 and −9, donors were stimulated with injection granulocyte colony-stimulating Factor (G-CSF), 7.5 *μ*g/kg BW/ day subcutaneously followed by BM aspiration from their posterior superior iliac crest under local anesthesia, in which 60 mL BM was collected on day −8.

### 3.2. Isolation of MSC from Adipose Tissue

The resected adipose tissue was transported to the lab in self-designed proliferation medium with Dulbecco's modified eagle's medium (DMEM, Sigma, USA) (high glucose), 20% human albumin (Reliance Life Sciences, India), Fibroblast growth factor: 2 ng/mL, 1% Sodium pyruvate, and appropriate antibiotics which included penicillin, streptomycin, cefotaxime, and fluconazol and minced with knife into tiny pieces in Collagenase type I (10 mg/10 mL) solution. The entire contents of the medium were processed in culture dish and after mincing they were placed in incubator at 37°C with shaker arranged with 35 RPM for 1 hour, and subsequently transferred to 15 mL centrifuge tubes and centrifuged at 780 RPM for 8 minutes. After centrifugation the supernatant and pellets were separately cultured in proliferation medium on 100 sq.cm and 25 sq.cm cell+ plates (Sarstedt, USA), respectively, at 37°C with 5% CO2 under humid conditions for 10 days. Medium was replenished every other day.

### 3.3. Culture and Differentiation of h-AD-MSC into Insulin-Secreting Cells

On the 10th day of culture in proliferation medium, the cells were washed in phosphate buffered saline (1 N). The cells were harvested by means of trypsinization (0.25% Trypsin EDTA solution, Hi Media, India) and checked for viability using trypan blue, sterility (Bactec, USA) and counts in modified Neubauer chamber. For flow cytometric analysis of cells, CD 45(Per CP) negative and CD90 (PE)/CD73 (PE) (Becton, Dickinson, USA) positive tests were carried out. They were also stained by Giemsa and further subjected to differentiation in to insulin-secreting cells using differentiation medium with DMEM (glucose- 17.5 mM), DMEM: F 12, Nicotinamide, Activin A, Exendin 4, Pentagastrin, hepatocyte growth factor, B-27, N-2 serum supplement, and antibiotics. This cocktail upregulates gene expression, nourishes the cells, and prevents their further proliferation. No xenogenic material was used. 

The cells were kept in this medium for 3 days for differentiation and then subjected to isolation on Ficoll Hypaque by density gradient.

### 3.4. Testing for Molecular Marker Characterization of Insulin Secreting MSC

Cell pellet was then diluted with equal amount of medium and after testing for sterility, viability, and cell counts, subjected to immunofluorescence test for expression of transcription factors, paired box 6 (Pax-6), marker for glucagon production, isl-1, key regulator for normal islet cell development, which is the gene upregulating expression of insulin, and pancreatic duodenal homeobox gene (pdx-1) which is the regulator of *β*-cell specific gene expression, function, and for self-renewal of *β* progenitor cells [5, 6].

**C-Peptide and Insulin**

from supernatant of cultured cells were measured by chemiluminescence.

### 3.5. Glucose Challenge Assay

Cells were further incubated in 6 well plates at the concentration of 5 cells/cm sq. without glucose, in glucose, (90 mM), 5 mL, and 10 mL respectively for 2 hours and insulin and c-peptide levels were measured at the end.

### 3.6. Culture of BM

The aspirated BM was subjected to invitro expansion under self-designed medium using DMEM:F12 (1 : 1) with 20% human albumin, Erythropoietin (V.H.B. Life Sciences, Inc, India), 10 *μ*L/100 mL, G-CSF (Gennova Biopharma, India), 10 *μ*L/100 mL, Mitomycin C, 2 *μ*L/100 mL, nonessential amino acids, 1 mL/100 mL, Ascorbic acid, 10 *μ*L/100 mL, and antibiotics in CO2 incubator at 37°C with 5% CO2 under humid conditions. No xenogenic material was used. Medium was replenished every other day for 8 days.

### 3.7. Recipient Conditioning

Nonmyelo-ablative low-intensity conditioning included target specific irradiation to subdiaphragmatic lymph nodes, spleen, part of pelvic bones, and lumbar vertebrae (200 cGY × 5 days) from day −8 to −3 of transplantation. Anti-T cell antibody, 1.5 mg/kg BW and anti-B cell antibody, 6 mg/kg BW were administered intravenously on days –2 and −1, respectively, to prevent rejection and facilitate grafting of transplanted cells. No immunosuppressive medication was used post-transplantation.

### 3.8. Cell Transplantation

Cell cocktail was transplanted into recipient on day 0 under general anesthesia as per our own technique of omental cannulation by mini-laparotomy in which a mid-line incision of 3 cm was made 5 cm above umbilicus, omental vein was cannulated using 20 guage needle and cells were infused at the rate of 6–8 mL/min. Omental vein was ligated with silk after infusion, hemostasis checked and wound closed with vicryl 2/0 stitches and subcuticular stitches were taken using 3/0 monocryl [7].

### 3.9. Posttransplant Patient Monitoring

Patients were monitored closely for 4 hourly blood sugar levels for 3 days after transplantation following 12 hourly monitoring for 1 week. Fasting and postprandial (PP) blood sugar (BS) levels were checked weekly for the 1st month and fortnightly for next the 2 months. Serum c-peptide levels were measured daily for the 1st week and with mixed meal tolerance weekly for the 1st month. Insulin administration was made on sliding scale with an objective of maintaining FBS <150 mg% and PPBS around 200 mg%. Glutamic acid decarboxylase (GAD) antibodies were monitored by ELISA technique (Euroimmun -Medizinische Labordiagnostika AG, UK) before and 3 months after infusion. Recipient monthly body weight, and number of diabetic ketoacidosis (DKA) episodes were monitored and evaluated before and after infusion. Glycosylated hemoglobin (Hb1Ac) (reference range: normal: <8.3%, good control: 8.3 to 9%, fair control: 9-10%, poor control: >10%, Erba diagnostics Mannheim, GmbH, Germany) levels were measured at 2-month intervals post-transplantation.

Key endpoints of study were morbidity, mortality, untoward side effects from stem cell transplantation, and changes in exogenous insulin requirements (daily dose/duration). Secondary endpoints were serum C-peptide levels with mixed-meal tolerance test at monthly intervals, GAD antibodies and Hb1Ac following stem cell transplantation.

### 3.10. Patients

Eleven patients (7 males, 4 females) with mean age 21.1 years (range: 13–43 years) with mean disease duration of 8.2 years (range:  1–24 years) were subjected to co-transplantation with IS-AD-MSC+HSCT. Seven out of 11 patients had associated DKA episodes (1 to 5 episodes). Mean Hb1Ac levels were 8.47% (range: 6.2–10.3%) and mean insulin requirement was 1.14 units/kgBW/day (range: 0.42–2.4 units per day).

## 4. Results

Donors were parents and siblings, in 4 patients cousin in 1, and uncles in 2 patients.

### 4.1. Stem Cells

**Figure 2:**
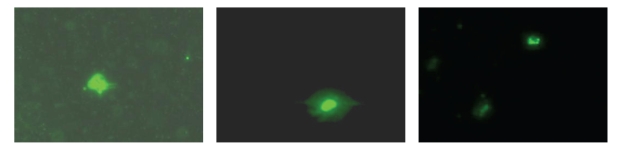
Indirect immunofluorescence demonstrating pax-6, isl-1, and pdx-1, from left to right, ×100.

Mean total cell quantum transplanted was 96.3 mL (range: 92 to 118 mL) with nucleated cell counts of CBM:  28 × 10^3^/*μ*L (range: 12.2 to 62.7 × 10^3^/*μ*L) and MSC- 1.2 × 10^3^/*μ*L (range: 0.5 to 2.1 × 10^3^/*μ*L), mean CD34^+^, 0.62% (range:  0.06 to 2.01%), mean CD 45^−^, 90^+^/73^+^ counts, and 39.99% (range: 16.6 to 81.4%)/30.1% (range: 14.1 to 65.7%). All of them expressed transcription factors pax-6, pdx 1, and isl-1 (Figure 2). Mean C-peptide level of cell inocula was 1.84 ng/mL (range: 1.15–3.6 ng/mL) and insulin level was 339.3 *μ* IU/L (range: 118 to 739 *μ* IU/L).

### 4.2. Patient Values

**Figure 3:**
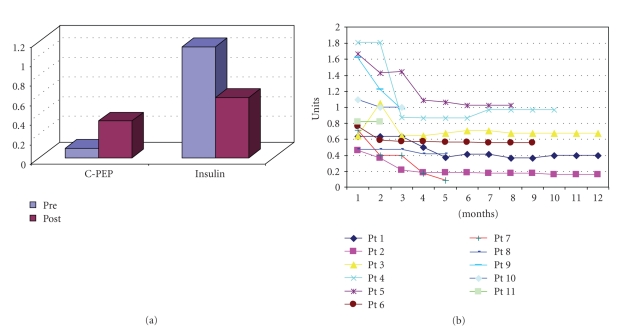
(a) Comparison of S.C-peptide levels and insulin requirement pre and post (7 months) stem cell transplantation. (b) Followup of exogenous insulin requirement (units/KgBW/Day).

Mean pretransplant serum C-peptide levels of 0.1 ng/mL (range: 0.02 to 0.3 ng/mL) increased gradually to mean 0.37 ng/mL (range: 0.1 to 1.8 ng/mL) (normal range:  0.7 to 1.9 ng/mL by Monobind Inc, USA) and mean pre-transplant exogenous insulin requirement of 1.14 units/kg BW/day (range: 0.42–2.1 units/kg BW/day) decreased to 0.63 units/kg BW/day (range: 0.09–1 unit/kg BW/day) (Figure 3(a)). It was observed that there was gradual fall in exogenous insulin requirement over the first 2 to 4 months which then remained steady (Figure 3(b)). Mean befor transplant Hb1Ac of 8.47% (range: 6.22 to 10.3%) decreased to 7.39% (range: 5.72 to 8.98%). 

GAD antibodies between 10 and 210 IU/mL in 5 patients decreased to values between 4 to 180 IU/mL and in 3 patients where values were >2000 IU/mL befor transplantation remained the same. No functional correlation was observed between insulin requirements/c-peptide levels and GAD antibody levels.

### 4.3. Statistical Analysis

Insulin requirement, Hb1Ac, and serum C-peptide levels were subjected to Student's paired t-test and change in insulin requirement was found to be the most significant with *P* = .009, Hb1Ac showed *P* = .03 and c-peptide values showed *P* = .05. 

There was no adverse/untoward side effect related to stem cell infusion or administration of induction therapy. Over a mean followup of 7.3 months (range: 2.2 months to 1 year) all patients continued to have a feeling of well-being after infusion and are physically more active, alert, on normal vegetarian diet, and better rehabilitated in their professional and personal lives. There was an impressive absence of DKA episodes in all of them. Pretransplant weight of 54.6 kg (range: 23.5 to 82 kg) increased marginally to 55.2 kg (range: 25 to 82 kg).

## 5. Discussion

Therapeutic strategies for addressing immune dysregulation of IDDM include nonactivating monoclonal antibodies against CD3, gene therapies, autologous HSCT, infusion of dendritic cells, T-regulatory lymphocytes, umbilical cord cells, embryonic or adult stem cells, and allogenic BM transplantation [4, 6]. Invitro rodent models have shown MSC derived from BM and spleen with capability of insulin-secretion to treat hyperglycemia [8]. Human BM-derived and adipose tissue-derived MSC have been found to be phenotypically identical cell populations as those of rodents [9]. We have generated in vitro MSC from human adipose tissue which qualify the definition standardized by the Mesenchymal and Tissue Stem Cell Committee of the International Society for Cellular Therapy. MSC have been defined as the cells having plastic-adherence when maintained under standard culture conditions, must have the ability of osteogenic, adipogenic and chondrogenic differentiation, must express CD73, CD90, and CD105, and must lack expression of hematopoietic lineage markers c-kit, CD14, CD34, CD 45, CD 11b, CD 29, CD79*α*, and HLA-DR [10]. Our cells fulfill these criteria. We further differentiated them to insulin secreting cells under defined culture conditions phenotypically identical to pancreatic *β* cells [11]. These cells expressed transcription factors pdx-1, pax-6, and isl-1, all three are central controlling genes capable of reprogramming nonpancreatic cells to surrogate *β* cell functions. Again our technique is a shortcut to reprogramming non-pancreatic cells as compared to vector-based gene transfer techniques [12]. 

Our results support the contention that combination of these three transcription factors represents the establishment of ectopic mechanisms to secrete insulin. These exciting results raise intriguing questions; whether this differentiation involves epigenetic reprogramming, or nuclear content of the MSC has become permissive to allow activation of this transcription program for *β* cell function. Whatever may be the reason this strategy has worked even in distantly related cells of origin.

## 6. Conclusion

This is the first report of successfully treating IDDM with co-transplantation of insulin-secreting adipose tissue derived MSC and HSCT. Easy and repeatable access to subcutaneous adipose tissue provides a clear advantage over isolation of MSC from BM. Isolation and culture techniques are simple and easy to perform.

## Figures and Tables

**Figure 1:**
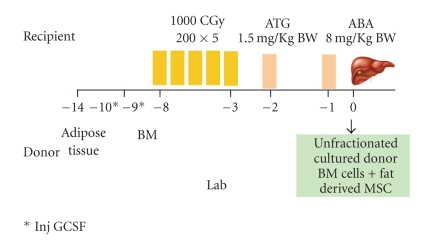
Ahmedabad paradigm of Cotransplantation of insulin secreting and hematopoietic stem cells for IDDM.

## Abbreviations

BM: Bone marrow
CBM: Cultured bone marrow
DKA: Diabetic keto acidosis
DM: diabetes mellitus
DMEM: Dulbecco's modified eagle's medium
GAD: glutamic acid decarboxylase
G-CSF: Granulocyte colony stimulating factor
Hb1Ac: Glycosylated hemoglobin
HSCT: Hematopoietic stem cell transplantation
IDDM: Insulin dependent diabetes mellitus
IRT: Insulin replacement therapy
IS-AD-MSC: Insulin secreting adipose tissue derived mesenchymal stem cells.
